# Atrial fibrillation associated with a thyroid stimulating hormone-secreting adenoma of the pituitary gland leading to a presentation of acute cardiac decompensation: A case report

**DOI:** 10.1186/1752-1947-2-67

**Published:** 2008-02-28

**Authors:** Jyothis T George, Jonathan C Thow, Bruce Matthews, Maurice P Pye, Vijay Jayagopal

**Affiliations:** 1 Department of Endocrinology, York Hospital, York, UK; 2 Department of Neurosurgery, Hull Royal Infirmary, Hull, UK; 3 Department of Cardiology, York Hospital, York, UK

## Abstract

**Introduction:**

Hyperthyroidism is a well established cause of atrial fibrillation (AF). Thyroid Stimulating Hormone-secreting pituitary tumours are rare causes of pituitary hyperthyroidism. Whilst pituitary causes of hyperthyroidism are much less common than primary thyroid pathology, establishing a clear aetiology is critical in minimising complications and providing appropriate treatment. Measuring Thyroid Stimulating Hormone (TSH) alone to screen for hyperthyroidism may be insufficient to appropriately evaluate the thyroid status in such cases.

**Case presentation:**

A 63-year-old Caucasian man, previously fit and well, presented with a five-day history of shortness of breath associated with wheeze and dry cough. He denied symptoms of hyperthyroidism and his family, social and past history were unremarkable. Initial investigation was in keeping with a diagnosis of atrial fibrillation (AF) with fast ventricular response leading to cardiac decompensation.

TSH 6.2 (Normal Range = 0.40 – 4.00 mU/L), Free T3 of 12.5 (4.00 – 6.8 pmol/L) and Free T4 51(10–30 pmol/L). Heterophilic antibodies were ruled out. Testosterone was elevated at 43.10 (Normal range: 10.00 – 31.00 nmol/L) with an elevated FSH, 18.1 (1.0–7.0 U/L) and elevated LH, 12.4 (1.0–8.0 U/L). Growth Hormone, IGF-1 and prolactin were normal. MRI showed a 2.4 cm pituitary macroadenoma. Visual field tests showed a right inferotemporal defect.

While awaiting neurosurgical removal of the tumour, the patient was commenced on antithyroid medication (carbimazole) and maintained on this until successful trans-sphenoidal excision of the macroadenoma had been performed. AF persisted post-operatively, but was electrically cardioverted subsequently and he remains in sinus rhythm at twelve months follow-up off all treatment.

**Conclusion:**

This case reiterates the need to evaluate thyroid function in all patients presenting with atrial fibrillation. TSH-secreting pituitary adenomas must be considered when evaluating the cause of hyperthyroidism. Early diagnosis and treatment of such adenomas is critical in reducing neurological and endocrine complications.

## Introduction

Atrial fibrillation (AF) is a common arrhythmia associated with increased morbidity and mortality [1] and its association with hyperthyroidism is well documented [2]. Thyroid function tests to exclude hyperthyroidism are part of the clinical assessment of patients presenting with AF [1]. However, the optimum method to assess thyroid function is not clearly defined. Measurement of Thyroid Stimulating Hormone (TSH) alone as a 'screening test' may result in the misdiagnosis of patients with pituitary causes of hyperthyroidism [3].

TSH-secreting pituitary tumours (TSHomas) are rare causes of hyperthyroidism where there is stimulation of the thyroid gland by TSH resulting in thyroid over-activity [4]. Failure to diagnose these correctly and inadvertent thyroid ablation in the case of TSHomas can result in tumour enlargement with neurological and endocrine complications [4]. Clinical features of hyperthyroidism are usually present at diagnosis; sometimes these are milder than expected given the level of thyroid hormones and also perhaps due to the longstanding duration of the hyperthyroidism. Acute presentation is uncommon [5].

## Case presentation

A 63-year-old Caucasian man, previously fit and well, presented with a five-day history of shortness of breath associated with wheeze and dry cough. His symptoms had progressively become worse, limiting his exercise tolerance. He denied symptoms of hyperthyroidism such as heat intolerance or weight loss and reported no palpitations or chest pain. His past history was unremarkable with no history of ischaemic heart disease or diabetes and he was taking no regular medications. He lived independently at home with his wife, working six days a week and had stopped smoking 30 years ago. His systemic enquiry and family history were unremarkable.

### Clinical examination

The patient had tachycardia, irregularly irregular, at 122/min with a raised jugular venous pressure (JVP) and a respiratory rate of 24/minute. He had no peripheral oedema or cyanosis. Cardiovascular auscultation revealed a soft mitral murmur. Baseline full blood count, electrolytes and renal function were normal. Chest X-ray showed cardiomegaly, pulmonary congestion and a small right pleural effusion.

### Initial diagnosis

A diagnosis of Atrial Fibrillation (AF) with fast ventricular response was made and the patient was given intravenous diuretics and digoxin and anticoagulation was commenced with Low Molecular Weight Heparin. Despite 48 hours of treatment, the tachycardia persisted and the JVP remained elevated. Echocardiogram, though limited by tachycardia, showed no impairment of ventricular function.

### Investigations

Thyroid Function Tests showed TSH 6.2 (Normal Range = 0.40 – 4.00 mU/L), Free T3 of 12.5 (4.00 – 6.8 pmol/L) and Free T4 51(10–30 pmol/L). Repeat Thyroid Function Tests showed TSH 6.6, Free T4 57 and Free T3 19.4 confirming a pituitary cause for hyperthyroidism. Retesting at a second laboratory corroborated these values. Heterophilic antibodies, which can interact with immunoassays, were ruled out. Pituitary function tests showed testosterone levels were elevated at 43.10 (Normal range: 10.00 – 31.00 nmol/L) along with elevated FSH, 18.1 (Normal range: 18.1 U/L) and elevated LH at 12.4 (1.0–8.0 U/L). Growth Hormone (GH), IGF-1 and prolactin were normal.

**Figure 1:**
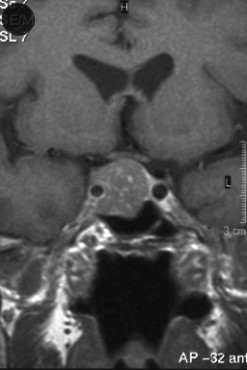
MRI of Pituitary – on admission.

An MRI of the brain showed a pituitary macroadenoma, 2.4 cm in diameter, abutting the optic chiasm, deviating the pituitary infundibulum and internal carotid artery. Visual field tests showed a defect in the right inferotemporal field with a normal field on the left. (Figure 1)

The patient was commenced on warfarin, but his International Normalised Ratio (INR) estimating his control on warfarin was markedly elevated with loading doses of warfarin and required reversal of anticoagulation with Vitamin K and introduction of low molecular weight heparin.

### Treatment and management

The patient was diagnosed as having a TSHoma and a decision was made to attempt neurosurgical removal. He was commenced on antithyroid medication (carbimazole) in the interim. Attempts to wean down his carbimazole resulted in an escalation of his symptoms and he was therefore maintained on this until successful trans-sphenoidal excision of his pituitary macroadenoma had been performed.

His AF persisted post-operatively, but was electrically cardioverted and he remains in sinus rhythm at twelve months follow-up off all treatment.

### Outcome

**Figure 2:**
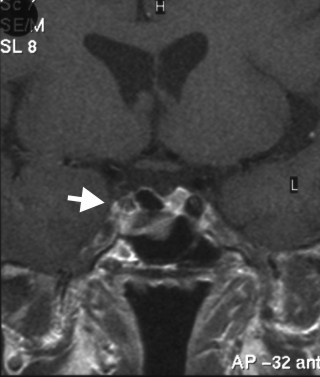
MRI of Pituitary – post operative.

Post-operative MRI showed near total removal of the pituitary adenoma. (Figure 2) Histology showed positive staining for GH, Prolactin and TSH. His TSH and T4 levels have remained normal post-operatively and glucagon stimulation test demonstrated adequate cortisol and GH response. His testosterone levels have also normalised after a transient fall post-operatively. GH, Prolactin and IGF-1 levels remained normal. FSH is persistently slightly raised at 9.1 U/L (1.0–7.0). The patient is asymptomatic, needing no treatment and back at full time employment. Arrangements are in place for a follow-up pituitary MRI.

## Discussion

TSH producing tumours of the pituitary are usually large tumours and they usually cause enlargement of the thyroid gland. Despite this however, overt thyrotoxicosis is uncommon, possibly due to a reduction in the biological activity of the secreted TSH. This case illustrates that although uncommon and thought to present with insidious symptoms, TSH secreting pituitary tumours (TSHomas) can occasionally present with acute manifestations like rapid atrial fibrillation and cardiac decompensation.

The majority of TSHomas (72%) secrete TSH alone, but 16% also secrete growth hormone (GH), 11% secrete prolactin (PRL), and 1% secretes LH or FSH [6]. The elevation in serum testosterone levels in our patient was secondary to co-secretion of LH and FSH by the adenoma. This illustrates the need to systematically assess complete pituitary function in such patients especially as some pituitary adenomas express positive histological staining for hormones without corresponding biochemical hyper-secretion [7].

Thyroid hormone concentrations can influence the metabolic rates of proteins and thus can alter the amount of vitamin K-dependent clotting factors, which in turn can alter the sensitivity to warfarin [8,9]. This may lead to changes in anticoagulation, increasing the risk of thromboembolic or hemorrhagic events. The volatile response to warfarin treatment in our patient stabilised with correction of his hyperthyroidism. Patients with AF associated with hyperthyroidism therefore need close monitoring of their anticoagulation profile.

It is critical to distinguish central hyperthyroidism from the more common types of primary hyperthyroidism. Measuring TSH alone as a 'screening test' may result in misdiagnosis of patients with TSHomas. Elevated Free T4 and/or T3 levels with a normal (non-suppressed) TSH should trigger investigations looking for a pituitary cause as inadvertent thyroid ablation resulting from such misdiagnosis can result in enlargement of these tumours [4]. Assay cross reactivity, presence of heterophilic antibodies and pituitary resistance to thyroid hormones are the three important alternatives to be considered. Assessment of TSH alpha subunit and TRH stimulation tests can help in differentiating TSHomas from pituitary and peripheral resistance to thyroid hormones. These tests were considered in our case but not performed as the diagnosis was adequately established from the available biochemistry and radiological appearance. We feel that further testing would not have contributed further in the evaluation of our patient but accept that it often can be useful in the diagnostic work up of such cases.

## Conclusion

Elevated Free T4 and/or T3 levels with a normal (non-suppressed) TSH should trigger a thorough investigation process looking for a pituitary cause of hyperthyroidism. In pituitary hyperthyroidism, complete pituitary function tests help identify pituitary adenomas that co-secrete other hormones along with TSH. Early diagnosis and treatment of TSH-secreting adenomas is critical in avoiding the neurological and endocrine complications that can result especially when misdiagnosed as primary hyperthyroidism and treated with radioiodine ablation.

This case also highlights the need to evaluate thyroid function in all patients presenting with atrial fibrillation. Presentation of TSH-secreting pituitary adenomas with acute cardiac decompensation is uncommon, but prompt management of the underlying hyperthyroidism is critical to a successful outcome.

## Abbreviations

AF = Atrial Fibrillation; FSH = Follicle Stimulating Hormone; GH = Growth Hormone; IGF-1 = Insulin-like Growth Factor-1; LH = Luteinising Hormone; TSH = Thyroid Stimulating Hormone; TSHoma = TSH Secreting Pituitary Adenoma; TRH = Thyrotropin Releasing Hormone.

## Competing interests

The author(s) declare that they have no competing interests.

## Authors' contributions

JCT, MPP and VJ were involved in the medical management of the patient. BM undertook surgical interventions on the patient. VJ and JTG identified educational opportunities in the case and obtained written informed consent from the patient. JTG carried out the literature search and produced the draft manuscript. All authors reviewed and approved the final manuscript.

## Consent

Written informed consent was obtained from the patient for publication of this case report and accompanying images. A copy of the written consent is available for review by the Editor-in-Chief of this journal.
